# Safety Profile of Molnupiravir in the Treatment of COVID-19: A Descriptive Study Based on FAERS Data

**DOI:** 10.3390/jcm12010034

**Published:** 2022-12-21

**Authors:** Greta Santi Laurini, Nicola Montanaro, Domenico Motola

**Affiliations:** 1 Unit of Pharmacology, Department of Medical and Surgical Sciences, Alma Mater Studiorum University of Bologna, 40126 Bologna, Italy; 2 The Alma Mater Studiorum University of Bologna, 40126 Bologna, Italy

**Keywords:** molnupiravir, COVID-19, safety, pharmacovigilance, FAERS

## Abstract

Concerns have been raised about the actual benefit and safety of molnupiravir, a new antiviral treatment for coronavirus disease 2019 (COVID-19). In order to provide additional evidence to support its use, we aimed to evaluate the real safety profile based on post-marketing pharmacovigilance data. Molnupiravir safety data were captured from the FDA Adverse Event Reporting System (FAERS). We performed a descriptive analysis of the baseline demographic characteristics of patients who experienced at least one adverse drug reaction (ADRs) related to molnupiravir, and then evaluated those most frequently reported. As of 31 March 2022, 612 reports of ADRs related to molnupiravir were submitted to the FDA, 301 (49.18%) were related to females and 281 (45.92%) to males. Most reports (524; 85.62%) were submitted by healthcare professionals and 345 (56.37%) concerned serious outcomes. The most common reported ADRs were diarrhoea (57; 4.51%), rash (36; 2.85), nausea (29; 2.30%), and COVID-19 pneumonia (22; 1.74%). The most frequent adverse reactions reported with molnupiravir in the U.S. post-marketing experience are consistent with the safety evaluation of the antiviral medicine. Even if no evident safety concerns emerged, an unexpectedly high rate of serious adverse reactions together with a few cases of potential new adverse reactions occurred.

## 1. Introduction

The coronavirus disease 2019 (COVID-19) pandemic, declared by the World Health Organization in March 2020, has so far resulted in more than 570 million confirmed cases and over 6 million deaths globally [1]. Although vaccination is still recommended as the best strategic response, safe and effective new treatments are needed for patients infected with the severe acute respiratory syndrome coronavirus 2 (SARS-CoV-2).

One of the latest tools in the COVID-19 therapeutic armamentarium has been the antiviral molnupiravir, also known under its brand name Lagevrio^®^. Originally synthesised at the Emory Institute for Drug Development (EIDD) to treat Venezuelan equine encephalitis virus (VEEV) [2], molnupiravir was developed by Merck Sharp & Dohme in collaboration with Ridgeback Biotherapeutics due to its demonstrated activity against SARS-CoV-2 [3,4].

Molnupiravir is an oral, small-molecule antiviral prodrug of N-hydroxycytidine (NHC), which inhibits viral replication through lethal mutagenesis. Once molnupiravir is converted to the active ribonucleoside analogue, NHC is phosphorylated intracellularly to NHC triphosphate and then incorporated by the SARS-CoV-2 RNA-dependent RNA polymerase (RdRp) into a viral genome. The resultant NHC containing RNAs misdirects the viral polymerase to incorporate either guanosine or adenosine, leading to an accumulation of deleterious mutations and thus to non-infectious, non-replicating viruses [3,4,5]. Because of molnupiravir-induced SARS-CoV-2 mutagenesis, theoretical concerns have been raised that molnupiravir may cause mutations in human DNA, especially in rapidly dividing human tissues [6]. However, based on the available data, regulatory authorities, including the FDA, concluded that molnupiravir has a low risk for mutagenicity and genotoxicity, and allowed its clinical use [7].

Molnupiravir’s promising clinical benefits are from the MOVe-OUT phase 3 trial [5], which evaluated its safety and efficacy in non-hospitalised, unvaccinated adults with laboratory-confirmed SARS-CoV-2 infection and at risk for severe illness. If started within 5 days of symptom onset, molnupiravir was found to be effective for the treatment of mild to moderate COVID-19, reducing the risk of progression to severe disease and thus providing global confidence in a new antiviral therapy.

Unlike monoclonal antibodies (mAbs) [8,9,10,11,12], which are another option for outpatients with COVID-19 and at risk of critical illness, molnupiravir is administered orally outside a medical setting, and is therefore readily and easily usable as soon as COVID-19 is diagnosed. Furthermore, while the currently available mAbs may have a loss of activity against new variants [13,14], molnupiravir has demonstrated to be a high barrier to the development of resistance [5,15,16]. As new variants of SARS-CoV-2 continue to emerge, molnupiravir is expected to be an important tool in the management of COVID-19.

Based on the compelling interim results of MOVe-OUT [17], the UK medicines regulator (Medicines and Healthcare Products Regulatory Agency, MHRA) issued the world-first conditional marketing authorisation of the antiviral drug molnupiravir on 4 November 2021 [18,19]. Once the full study was published [5], the Food and Drug Administration (FDA) also granted Emergency Use Authorization (EUA) for molnupiravir, but limited to situations where other FDA-authorised COVID-19 treatment options are not accessible or clinically appropriate [20]. The European Medicines Agency (EMA), on the other hand, has not yet formally approved molnupiravir, but only issued advice for countries considering its use prior to marketing authorisation [21].

Considering the important issues with the Merck’s MOVe-OUT clinical trial, many in the science community are questioning the use of molnupiravir in emergency settings. Since the full dataset of the study was published, several statistical and methodological limitations emerged, as well as concerns about the actual benefit of molnupiravir on improving clinical outcomes in non-hospitalised at-risk patients with COVID-19 [22,23,24,25,26,27].

Given the weakness of the current evidence supporting its widespread use, continuing to provide additional data on safety is critical for the ongoing evaluation of the benefit/risk profile. Post marketing pharmacovigilance is essential to obtain updated safety information for new drugs since premarketing clinical trials are not powered to detect it. Addressing this strong need, we collected all suspected adverse drug reactions (ADRs) reported in clinical practice following the administration of molnupiravir in the United States.

## 2. Materials and Methods

Molnupiravir safety data were captured from the FDA Adverse Event Reporting System (FAERS) using its web-based Public Dashboard [28]. It is a national database designed to collect reports of adverse events submitted to the FDA by the pharmaceutical industry, healthcare providers and consumers. As a post-marketing monitoring system, FAERS is a valuable tool for the safety surveillance of drugs and therapeutic biologic products after they are approved by the FDA. An Individual Case Safety Report (ICSR) in the FAERS database is identified by a unique Case ID and includes information such as patient characteristics, suspected and concomitant drugs, the date the case was received by the FDA, the occupation of the reporter and finally a description of the suspected adverse drug reactions (ADRs), including outcome and seriousness.

In the present post-marketing safety surveillance survey, we considered all reports of ADRs with molnupiravir as a suspected medicinal product in the period between 23 December 2021, the date molnupiravir had been authorised by the FDA for emergency use [20], and 31 March 2022. Given the possibility that the same report may be submitted by several reporters (e.g., by a consumer and by the sponsor), we firstly identified and ruled out any duplicates, so as to ensure that the same adverse event was not counted more than once. 

Based on the reports collected, we performed a descriptive analysis of the baseline demographic characteristics of patients who experienced at least one adverse event related to the antiviral drug (Table 1). All ADRs with reporting rate ≥3 occurring after administration of molnupiravir are listed in Table 2. We previously excluded events which were not to be considered as suspected adverse reactions (e.g., product use issue, wrong technique and accidental underdose). Moreover, for the most reported events, it was checked whether they were listed in the corresponding safety assessment and thus known before marketing [7].

## 3. Results

As of 31 March 2022, a total of 612 reports of ADRs related to molnupiravir had been submitted to the FDA. Among the individuals who experienced at least one adverse event after taking the antiviral, 301 (49.18%) were females and 281 (45.92%) were males. For a total of 30 people (4.90%), sex had not been listed on the safety reports. Most patients (241; 39.38%) belonged to the 65–85 years age class, while the remaining ones were aged >85 (119; 19.44%) or between 18 and 64 years old (164; 26.80%). Only three reports were related to 17-year-old patients of unknown sex. Most of the reports of suspected ADRs were submitted by healthcare professionals (524; 85.62%) and a total of 345 (56.37%) concerned serious outcomes. Overall, 1263 adverse events were reported as suspected side effects associated with molnupiravir. The top five most common reported ADRs were COVID-19 (62 events; 4.91%), diarrhoea (57; 4.51%), rash (36; 2.85), nausea (29; 2.30%), and COVID-19 pneumonia (22; 1.74%). Among patients treated with molnupiravir, 183 (29.90%) were concomitantly taking other products, mainly acetaminophen, amlodipine besylate, magnesium oxide, carbocysteine, dextromethorphan hydrobromide, and aspirin.

## 4. Discussion

This U.S. surveillance survey aims to provide preliminary findings on the safety of molnupiravir in a real clinical setting. Although no evident safety concerns have emerged during the MOVe-OUT clinical trial [5], the safety profile is limited by a small sample of unvaccinated adults with COVID-19. As more patients gained access to the drug, post-approval pharmacovigilance activities became critical for the detection of possible new adverse drug reactions.

As of 31 March 2022, three months after the FDA authorised molnupiravir for emergency use, a total of 612 individuals in the United States experienced at least one adverse event after receiving the new antiviral therapy. Based on the collected data, most patients were over 64 years of age (58.8%). Although predisposition seems to be multifactorial for most reactions, age is known to have a critical impact on the occurrence of ADRs [29]. In older people, the risk of adverse drug reactions is higher for several reasons, including age-related changes in pharmacokinetics and pharmacodynamics, the increasing prevalence of comorbidity, polypharmacy, and inappropriate prescribing [30]. Another well-documented factor that affects the development of ADRs is concomitant diseases, regardless of the age of the patient [29]. Given that molnupiravir is recommended in the elderly, as well as in those with pre-existing medical conditions, considering the high risk for both to develop severe illness from COVID-19, all patients eligible for treatment are more vulnerable to the development of adverse reactions.

Among reports of adverse events submitted to the FDA, a total of 345 (56.4%) concerned serious outcomes, including 70 deaths (11.4%). In the phase 3 MOVe-OUT trial, ADRs were reported as serious in 49 of 216 participants (22.7%) in the molnupiravir group with at least one adverse event, resulting in two deaths overall (0.9%) [5]. Since the proportion of patients with serious outcomes is significantly higher in our survey, it is possible that an unexpected pattern of serious ADRs has emerged with more widespread use. However, since many serious adverse events were reported as COVID-19 related, it may also be that molnupiravir was only partially effective in preventing disease progression, raising further concerns about the actual clinical benefit. Although the latest data from the phase 3 component of MOVe-OUT suggests additional benefits on other clinically relevant outcomes, such as respiratory interventions and medical services [31], the issue concerning the primary efficacy end point as a reduction in the risk for hospitalisation or death due to COVID-19 progression still remains unclear.

In the first quarter of marketing in the United States, the adverse reactions related to molnupiravir with the highest number of reports were COVID-19, diarrhoea, rash, nausea, COVID-19 pneumonia, vomiting, and dizziness. The item COVID-19 lends itself to different interpretations. One may argue that it is simply a writing mistake of the reporters, with COVID-19 being the target disease for the antiviral treatment rather than an adverse event. On the other hand, it cannot be excluded that the mention of the target disease in the field of adverse events may be understood as a statement of therapeutic failure, as well as the term COVID-19 pneumonia. The high number of either term in our list makes this second interpretation preferable. The pattern of ADRs is consistent with the safety assessment supporting the Emergency Use Authorization [7]. Based on the phase 3 MOVe-OUT study, diarrhoea, nausea and dizziness were identified as the most common adverse reactions related to the molnupiravir treatment [5], while rash was included among skin and subcutaneous tissue disorders after being observed during post-authorisation experience [7]. Otherwise, COVID-19 pneumonia and several other outcomes reported as adverse reactions (e.g., pyrexia, oxygen saturation decreased, asthenia, dyspnoea, cough) are likely deemed to be related to the clinical presentation of SARS-CoV-2 infection, ranging from mild to critical illness [32,33,34]. According to the National Institutes of Health (NIH) COVID-19 Treatment Guidelines [35], patients with mild COVID-19 present with fever, cough, malaise, fatigue, asthenia, headache, nausea, vomiting, diarrhoea, and any general symptoms of a viral infection. COVID-19 may then progress into moderate or severe disease by evidence of lower respiratory disease, including dyspnoea, decreased oxygen saturation and pneumonia, all of which were widely observed in our analysis.

Although none of the most common adverse reactions above gave cause for concern, some outcomes of special interest (e.g., seizure, renal impairment, arrhythmia, cardiac arrest and cardiac failure) have occurred in clinical practice less frequently. Particularly noteworthy are the four cases of renal impairment, which are consistent with the recent case report by Hasan Esat Yücel of a 67-year-old patient who developed severe vomiting and diarrhoea due to molnupiravir and progressed to severe kidney function deterioration [36]. Even though no clinically meaningful abnormalities of kidney or brain or cardiac function were found in the clinical evaluation of molnupiravir [5], some rare adverse reactions may have emerged with the widespread use of the investigational drug. As COVID-19 affects not only the respiratory system but also other organs such as the kidneys, brain and heart [32,37,38,39,40], a closer monitoring is required to assess a possible link between molnupiravir therapy and such extrapulmonary manifestations.

The present post-marketing safety surveillance survey should be considered in light of the following limitations. Since the reports of adverse reactions were captured from the U.S. national passive reporting system, these may be incomplete, inaccurate, and of poor quality, making it difficult to detect duplicates and thus distorting the frequency of ADRs. Moreover, as the total number of patients treated with molnupiravir is unknown, we were not able to establish the occurrence rates of the events, but could only provide a frequency estimate over the total number of outcomes submitted to FAERS [28]. Under-reporting, on the other hand, which is one of the main limitations of all passive surveillance systems including FAERS, is expected to be low since health care providers are responsible for mandatory reporting of all serious adverse reactions potentially related to molnupiravir [7]. Since the present survey focused on the occurrence of adverse events related to molnupiravir only, monitoring of adverse events caused by potential drug–drug interactions is needed, including those resulting from the combination treatment of molnupiravir with remdesivir [41].

In conclusion, even if our safety surveillance survey was unable to establish a causal relationship between molnupiravir therapy and any of the reported adverse reactions, it provides preliminary findings on the safety profile during clinical practice, adding valuable information for the evaluation of the benefit/risk profile.

## 5. Conclusions

The most common adverse reactions reported with molnupiravir in the U.S. post-marketing experience are consistent with the safety evaluation of the antiviral medicine. An unexpectedly high rate of serious adverse reactions together with a few cases of potential new adverse reactions occurred. As the use of this new therapy increases, continued monitoring is required for a better assessment of the real safety profile.

## Figures and Tables

**Table 1:** Patients’ age and sex who expressed at least one adverse event related to molnupiravir.

Age	Sex	Subtotal	%	Total	%
12–17 years	Female	0	0.00	3	0.49
	Male	0	0.00		
	Unknown	3	0.49		
18–64 years	Female	79	12.91	164	26.80
	Male	84	13.73		
	Unknown	1	0.16		
65–85 years	Female	112	18.30	241	39.38
	Male	126	20.59		
	Unknown	3	0.49		
85+ years	Female	68	11.11	119	19.44
	Male	46	7.52		
	Unknown	5	0.82		
Not Specified	Female	42	6.86	85	13.89
	Male	25	4.08		
	Unknown	18	2.94		
Total		612	100	612	100

**Table 2:** Most reported adverse events in VAERS.

Adverse Events	N	%
COVID-19	62	4.91
Diarrhoea	57	4.51
Rash	36	2.85
Nausea	29	2.30
COVID-19 Pneumonia	22	1.74
Maternal Exposure During Pregnancy	22	1.74
Vomiting	22	1.74
Dizziness	21	1.66
Pneumonia	19	1.50
Drug Ineffective	17	1.35
Urticaria	16	1.27
Pyrexia	16	1.27
Feeling Abnormal	15	1.19
Oxygen Saturation Decreased	13	1.03
Death	13	1.03
Pneumonia Aspiration	12	0.95
Decreased Appetite	12	0.95
Abdominal Pain	12	0.95
Headache	12	0.95
Condition Aggravated	10	0.79
Drug Eruption	9	0.71
Dehydration	9	0.71
Dysphagia	9	0.71
Pruritus	9	0.71
Asthenia	8	0.63
Dyspnoea	7	0.55
Erythema	7	0.55
Syncope	7	0.55
Malaise	6	0.48
Loss Of Consciousness	6	0.48
Haematochezia	5	0.40
Cough	5	0.40
Seizure	5	0.40
Tremor	5	0.40
Pain	5	0.40
Feeding Disorder	4	0.32
Depressed Level Of Consciousness	4	0.32
Bradycardia	4	0.32
Hypotension	4	0.32
Pancytopenia	4	0.32
Insomnia	4	0.32
Renal Impairment	4	0.32
Cardiac Arrest	4	0.32
Eczema	3	0.24
International Normalised Ratio Increased	3	0.24
Rash Pruritic	3	0.24
Melaena	3	0.24
Sepsis	3	0.24
Pneumonia Bacterial	3	0.24
Pallor	3	0.24
Flushing	3	0.24
Wheezing	3	0.24
Deafness Unilateral	3	0.24
Altered State Of Consciousness	3	0.24
Burning Sensation	3	0.24
Hallucination	3	0.24
Rash Erythematous	3	0.24
Respiratory Disorder	3	0.24
Off Label Use	3	0.24
Vision Blurred	3	0.24
Blood Pressure Increased	3	0.24
Arrhythmia	3	0.24
Hepatic Function Abnormal	3	0.24
Hypertension	3	0.24
Neutropenia	3	0.24
Fatigue	3	0.24
Hyperhidrosis	3	0.24
Palpitations	3	0.24
Abdominal Pain Upper	3	0.24
Chest Pain	3	0.24
Abdominal Distension	3	0.24
Cardiac Failure	3	0.24
Pain In Extremity	3	0.24

## Data Availability

The data that support the findings of this study are available from the corresponding author upon reasonable request.
